# Soybean oil increases SERCA2a expression and left ventricular contractility in rats without change in arterial blood pressure

**DOI:** 10.1186/1476-511X-9-53

**Published:** 2010-05-26

**Authors:** Eduardo Hertel Ribeiro, Aurélia Araújo Fernandes, Eduardo Frizzera Meira, Priscila Rossi Batista, Fabiana Dayse Magalhães Siman, Dalton Valentim Vassallo, Alessandra Simão Padilha, Ivanita Stefanon

**Affiliations:** 1 Department of Physiological Sciences, Federal University of Espirito Santo, Vitoria, Brazil; 2 Department of Physiological Sciences, EMESCAM, Vitoria, Brazil

**Keywords:** Perfuse Heart, Coronary Perfusion Pressure, Inotropic Response, Myosin ATPase Activity, SERCA2a Expression

## Abstract

**Background:**

Our aim was to evaluate the effects of soybean oil treatment for 15 days on arterial and ventricular pressure, myocardial mechanics and proteins involved in calcium handling.

**Methods:**

Wistar rats were divided in two groups receiving 100 μL of soybean oil (SB) or saline (CT) i.m. for 15 days. Ventricular performance was analyzed in male 12-weeks old Wistar rats by measuring left ventricle diastolic and systolic pressure in isolated perfused hearts according to the Langendorff technique. Protein expression was measured by Western blot analysis.

**Results:**

Systolic and diastolic arterial pressures did not differ between CT and SB rats. However, heart rate was reduced in the SB group. In the perfused hearts, left ventricular isovolumetric systolic pressure was higher in the SB hearts. The inotropic response to extracellular Ca^2+^ and isoproterenol was higher in the soybean-treated animals than in the control group. Myosin ATPase and Na^+^-K^+^ATPase activities, the expression of sarcoplasmic reticulum calcium pump (SERCA2a) and sodium calcium exchanger (NCX) were increased in the SB group. Although the phosfolamban (PLB) expression did not change, its phosphorylation at Ser^16^ was reduced while the SERCA2a/PLB ratio was increased.

**Conclusions:**

In summary, soybean treatment for 15 days in rats increases the left ventricular performance without affecting arterial blood pressure. These changes might be associated with an increase in the myosin ATPase activity and SERCA2a expression.

**Electronic supplementary material:**

The online version of this article (doi:10.1186/1476-511X-9-53) contains supplementary material, which is available to authorized users.

## Introduction

Soybean oil and other oils rich in polyunsaturated fatty acids (PUFAs) are thought to be beneficial to cardiovascular health [1–3]. Their effects can be attributed to the presence of alpha-linolenic acid, an essential n-3 fatty acid found in vegetable cooking oils such as soybean and canola oils. In fact, a previous report suggested that both dietary and adipose levels of alpha-linolenic acid were associated with a large reduction in the risk of nonfatal acute myocardial infarction [1–3] and low consumption of alpha-linolenic acid has been correlated with an increase of coronary heart disease and sudden death of cardiac origin [4].

Alpha-linolenic acid can not be produced in the body and must be taken in by food. In both animals and humans, alpha-linolenic acid is desaturated and elongated into eicosapentaenoic (EPA) and docosahexaenoic (DHA) acid. It is also incorporated into plasma and tissue lipids, and its conversion is affected by the levels of linoleic acid.

McLennan [5] reported that when the major dietary fat was sunflower seed oil (77% linoleic acid) there was a 70% reduction in fatal ventricular fibrillation when coronary arteries were ligated compared with diets high in saturated or monounsaturated fats. Soybean oil contains over 50% linoleic acid. Therefore, it could contribute to the cardiac beneficial effects of soybean oil.

Pepe and McLennan [6] showed that fish oil improves postischemic recovery of cardiac contractile function. In their study, EPA and DHA were attributed as the primary causes for the efficacy of fish oil. Other sources of alpha-linoleic acid include canola, soybeans, and some fish including salmon. However, the mechanisms by which soybean oil exerts its cardiac protective effects have not yet been elucidated. Therefore, the goal of the present study was to investigate the effects of 15 days of soybean oil treatment in young healthy male rats on arterial and left ventricular pressure, left ventricular function of isolated perfused hearts, the Na^+^-K^+^ATPase (NKA), myosin ATPase activities and the expression of calcium handling proteins.

## Material and methods

### Animals and soybean oil treatment

Male 12-week old Wistar rats were used. Rats were housed at constant room temperature, humidity and light cycles, (12 h light/dark), with free access to tap water and were fed standard rat chow ad libitum. Care and use of laboratory animals and all experiments were in compliance with the guidelines for biomedical research, as stated by the Brazilian Societies of Experimental Biology and approved by the local ethics committee (EMESCAM -003/2007).

Rats were divided in two groups: soybean oil treatment (SB) and control (CT). The treated group received a daily dose of 0.1 mL (i.m.) of soybean oil for 15 days and the control group received a similar volume of 0.9% NaCl.

### Arterial blood pressure and left ventricular pressure measurements

At the end of treatment, rats were anesthetized with urethane (1.2 g/kg) and a polyethylene catheter (PE50) filled with heparinized saline (50 U/mL) was introduced into the carotid artery to measure the arterial systolic blood pressure (SBP) and diastolic blood pressure (DBP). The carotid artery catheter was introduced into the left ventricle to measure the systolic pressure (LVSP) and its positive and negative first derivatives (dP/dt max LV and dP/dt min LV, respectively), as well as the left ventricular end diastolic pressure (LVEDP). Following this procedure, the catheter was withdrawn from the LV and the arterial pressure was measured again to determine if damage to the aortic valve had occurred. The animal was discarded if a decrease in the diastolic blood pressure was observed.

### Isolated heart perfusion

After hemodynamic measurements, rats were treated with heparin (500 UI, i.p.) and after 10 minutes the heart was excised and mounted in an isolated organ chamber. The heart was then perfused according to the Langendorff technique at constant flow (10 mL/min) with Krebs bicarbonate buffered solution containing (in mM): 120 NaCl, 5.4 KCl, 1.25 CaCl_2_, 2.5 MgSO_4_, 1.2 Na_2_SO_4_, 2.0 NaH_2_PO_4_, 20 NaHCO_3_ and 11 glucose. This solution was continuously bubbled with 95% O_2_ and 5% CO_2_ (pH = 7.4) at 31°C. After mounting, the left atrium was opened and a soft distensible latex balloon mounted at the tip of a rigid plastic tube was inserted into the left ventricular cavity through the atrioventricular valve. The balloon was connected, via a Y piece, to a pressure transducer (TSD104A) and a syringe so that the diastolic pressure (DP) of the left ventricle could be adjusted to predetermined values by injecting water into the balloon. The developed left ventricular isovolumic systolic pressure (LVISP) was recorded using the pressure transducer (TSD104A) connected to the Biopac System (MP100; CA). The hearts were driven with isolated suprathreshold rectangular pulses (5 ms duration) at a baseline constant rate of 200 bpm using a pair of Ag/AgCl electrodes attached to the upper region of the right ventricle. All measurements began 1 h after mounting to allow the beating preparation to adapt to the in vitro conditions. The coronary perfusion pressure (CPP) was continuously recorded by connecting a pressure transducer (TSD104A) to the aortic pressure tube. Since coronary flow was kept constant (10 mL/min), changes of the CPP were dependent on changes of coronary resistance. Ventricular function curves were obtained by measuring the LVISP that developed while DP was increased from 0 to 30 mm Hg in 5 mm Hg steps.

To analyze the force-frequency relationship, the frequency was gradually increased from 200 to 350 bpm in 50 bpm steps. To analyze the inotropic response to Ca^2+^, the diastolic pressure was set at 10 mmHg and the steady-state LVISP was measured by increasing extracellular Ca^2+^ concentrations (0.62; 1.25; 1.87; 2.5; and 3.12 mM). Finally, a single dose of isoproterenol (100 μL, 10^-5^ M) was administered to evaluate the β-adrenoceptor response.

### Cardiac myosin ATPase activity

#### Preparation of tissue

Myosin was prepared from minced and homogenized left ventricles extracted briefly with KCl-phosphate buffer (0.3 M KCl and 0.2 M phosphate buffer) pH = 6.5. After precipitating myosin using a 15-fold dilution with water, the muscle residue was separated by filtration using cheesecloth. This procedure precipitates fragments of cells, including the membranes. The myosin-containing filtrate was centrifuged at 10,000 × g for 40 min. The precipitate was re-dissolved in phosphate buffer and 0.6 M KCl to elute the myosin under high ionic strength, and 1 mL of water was added for each gram of tissue to generate a new precipitate. The material was again centrifuged at 10,000 × g for 40 min and the muscle residue was separated by filtration. The material was re-dissolved again in 14 mL of water per gram of tissue, centrifuged, and filtered as before. The precipitate was dissolved in 50 mM N-(2-hydroxuethylpiperazine-N'-[2-ethanesulfonic acid]) sodium salt (HEPES pH 7) and 0.6 M KCl plus 50% glycerol (v/v) and stored at -20°C. Stored myosin was diluted in water (1:12) and centrifuged at 10,000 × g for 40 min. The precipitate was re-suspended in 50 mM HEPES (pH 7) and 0.6 M KCl and centrifuged at 600 × g for 5 min. The supernatant was used for enzyme assays.

### Assay to determine myosin ATPase activity

Myosin ATPase activity was determined by measuring inorganic phosphate (Pi) liberation from 1 mM ATP in the presence of 50 mM HEPES (pH 7), 0.6 M KCl, 5 mM CaCl_2_, or 10 mM ethylene glycol-bis (β-amino ethyl ether)-N,N,N',N'-tetra acetic acid (EGTA) in a final volume of 200 μL. ATP was added to the reaction mixture and pre-incubated for 5 minutes at 30°C. The reaction was initiated by adding the enzyme fraction (3-5 μg protein) to the reaction mixture. Samples were assayed in duplicate or triplicate and corrected for non-enzymatic hydrolysis by using controls assayed in the same conditions, except that the protein sample was added after interruption of the reaction using 200 μL of 10% trichloroacetic acid. The reaction was initiated by the addition of the protein sample to avoid the inactivation at 30°C caused by the lack of substrate. Pi concentration was determined by a previously described method [7]. The specific activity was reported as nmol Pi released per min per mg of protein. Protein concentration was measured according to the Bradford method [8] using bovine serum albumin as the standard.

### Na^+^-K^+^ ATPase activity

The enzymatic material was extracted as previously described [9]. Briefly, ventricular tissue was homogenized in a solution containing 20 mM Tris-HCl and 1 mM EDTA, pH 7.5. The homogenized tissue was centrifuged at 10,000 g for 20 minutes and precipitate was discarded. The supernatant was centrifuged at 10,500 g for one hour. The precipitate was resuspended in 20 mM Tris-HCl and 1 mM EDTA, pH 7.5 in a final volume of 400 μL.

Na^+^-K^+^ ATPase activity was assayed by measuring Pi liberation from 3 mM ATP in the presence of 125 mM NaCl, 3 mM MgCl_2_, 20 mM KCl, and 50 mM Tris-HCl (pH 7.5). The enzyme was preincubated for 5 minutes at 37°C and the reaction was initiated by adding ATP. Incubation times and protein concentration were chosen to ensure the linearity of the reaction. The reaction was quenched by the addition of 200 μL of 10% trichloroacetic acid. Controls with addition of the enzyme preparation after addition of trichloroacetic acid were used to correct for nonenzymatic hydrolysis of the substrate. All samples were examined in duplicate. The specific activity was reported as nmol Pi released per min per mg of protein unless otherwise stated. The specific activity of the enzyme was determined in the presence and absence of 5 mM ouabain.

### Western blot analysis

Proteins from homogenized hearts (50 μg for phosfolamban (PLB) and 100 μg for the other proteins) were separated by SDS-PAGE. Proteins were transferred to nitrocellulose membranes and incubated with mouse monoclonal antibodies for sarcoplasmic reticulum calcium pump (SERCA2a) (1:500, Affinity BioReagents, CO, USA), Na^+^Ca^2+^ exchange (NCX) (1:200, Abcam Cambridge, MA, USA), PLB (1 μg/ml, Affinity BioReagents, CO, USA), α1 of the Na^+^-K^+^ ATPase (NKA) (1:1000, Upstate Massachusetts, USA), α2 of the NKA (1:1000, Upstate Massachusetts, USA) and rabbit polyclonal antibodies for PLB phospho-Ser16 (1:5000, Badrilla, Leeds, UK). After washing, membranes were incubated with anti-mouse or anti-rabbit (1:5000, StressGen, Victoria, Canada) immunoglobulin antibody conjugated to horseradish peroxidase. After washing, immunocomplexes were detected using an enhanced horseradish peroxidase/luminal chemiluminescence system (ECL Plus, Amersham International, Little Chalfont, UK) and film (Hyperfilm ECL International). Signals on the immunoblot were quantified using the National Institutes of Health Image V1.56 computer program. As a loading control, the same membrane was used to determine GAPDH expression using a mouse monoclonal antibody (1:5000, Abcam Cambridge, MA, USA).

### Drugs and chemicals

Salts and reagents used, when not otherwise indicated, were purchased from Sigma and Merck (Darmstadt, Germany).

### Statistical analysis

The results are presented as mean ± SEM, with "N" indicating the number of observations. Values were analyzed using the t-test or ANOVA (one-and two-way). When ANOVA revealed a significant difference, the Tukey test was applied. p < 0.05 was considered significant.

## Results

At the end of the treatment, the body weight of animals treated with soybean oil was similar to controls (SB: 346 ± 5.5 vs. CT: 347 ± 7.2 g). Soybean oil treatment did not cause significant alterations in the left ventricle to body weight ratio compared to controls (SB: 1.91 ± 0.06 vs. CT: 1.92 ± 0.2 mg/g), suggesting that ventricular hypertrophy did not occur.

### Hemodynamic measurements

Systolic and diastolic arterial pressures did not differ between control and treated rats (Table 1). The heart rate was lower in the soybean oil group when compared with the control group. LVSP, LVEDP, dP/dt max LV and dP/dt min LV from treated rats did not differ when compared to controls (Table 1).

**Table 1:** Changes in systolic (SBP) and diastolic blood pressure (DBP), heart rate (HR), left (LVSP) ventricle systolic pressure, left ventricle diastolic pressure (LVEDP), and the positive (dP/dt max) and negative first time derivatives (dP/dt min) from control (CT) and soybean oil-treated rats (SB).

	CT (n = 11)	SB (n = 6)
**SBP (mm Hg)**	103 ± 4.10	94 ± 4.11
**DBP (mm Hg)**	60 ± 4.06	56 ± 3.63
**HR (bpm)**	315 ± 13	270 ± 12*
**LVSP (mm Hg)**	113 ± 4.01	118 ± 5.35
**LVEDP (mm Hg)**	4.38 ± 0.53	6.0 ± 0.44
**dP/dt**_**max**_**LV (mm Hg/sec)**	5045 ± 289	4401 ± 451
**dP/dt**_**min**_**LV (mm Hg/sec)**	-5436 ± 225	-5112 ± 314

Student "t" test, *p < 0.05 vs Control.

### Ventricular performance

**Figure 1:**
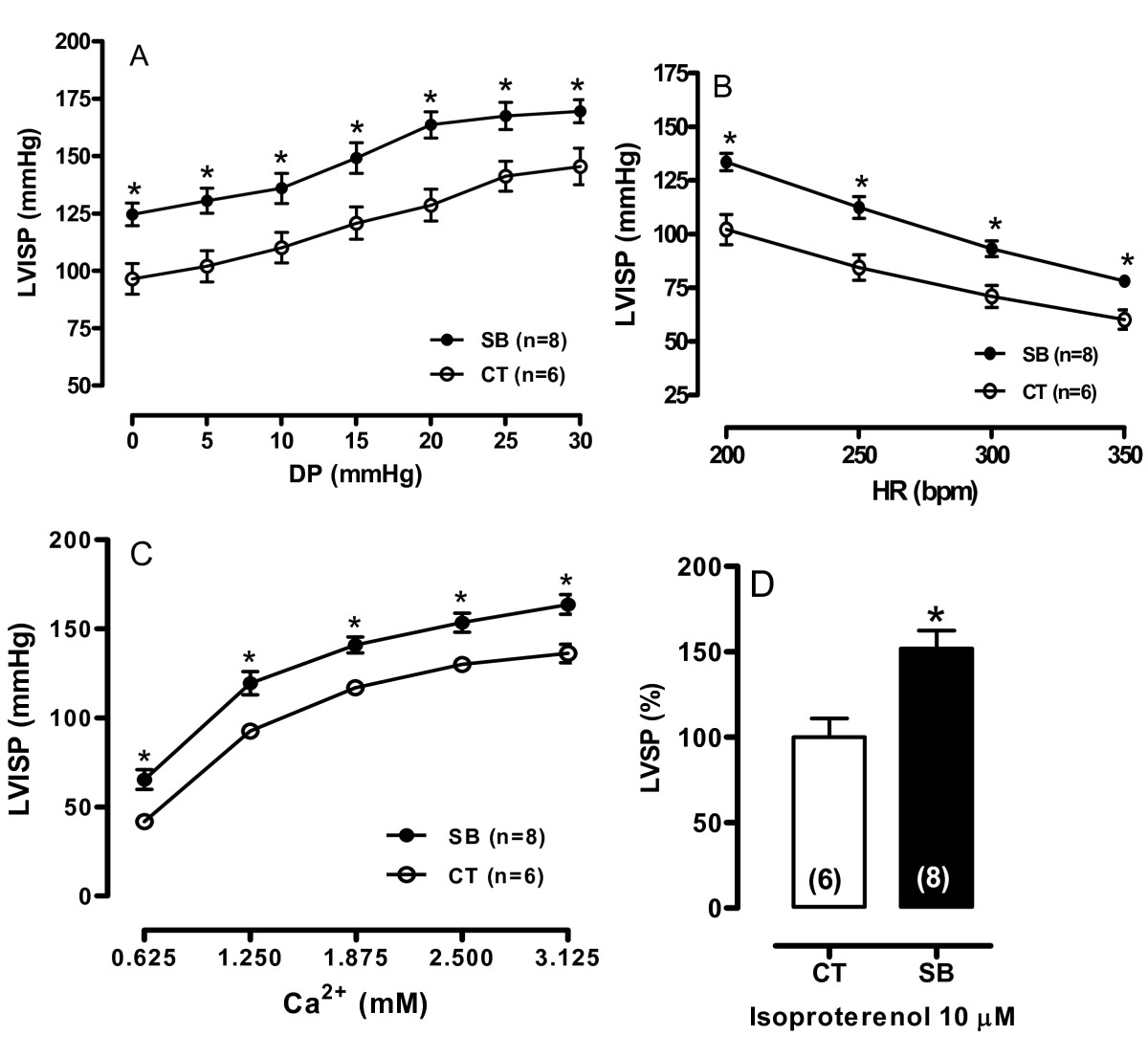
**(A) LVISP-Left ventricle isovolumetric systolic pressure curves obtained at different diastolic pressures (DP)**. (B) LVISP upon changes in heart rate stimulation. (C) Left ventricular curves were normalized to the DP of 10 mm Hg. Hearts were perfused using different extracellular Ca^2+^ concentrations. (D) β-adrenergic activation in the Langendorff apparatus (% of Control). Data are shown as mean ± SEM. *p < 0.05. ANOVA two-way and Tukey test. Number of animals used is indicated in parentheses.

Induction of the Frank-Starling mechanism was used to investigate the intrinsic inotropic response of the cardiac muscle of rats after soybean oil treatment. In the perfused hearts, LVISP was higher in the SB hearts, and the increment of DP from 0 to 30 mm Hg produced an increased inotropic effect in both groups (Figure 1A). Pressure derivatives showed a similar behavior as the ventricular pressure in the soybean oil-treated group. CPP did not differ between the groups.

To investigate if soybean oil treatment affected the action of inotropic interventions, heart rate and extracellular Ca^2+^ (0.62-3.12 mM) increment were examined. As expected, in the rat myocardium the increase of the rate of stimulation reduced LVISP in both groups (Figure 1B). However, LVISP was higher in the soybean oil group for all rate changes when compared to the control group.

The extracellular Ca^2+^ increment increased the LVISP in both groups, however in the SB treated rats this increment was enhanced when compared to the control group (Figure 1C).

To evaluate the β-adrenergic response, isoproterenol (10 μM) was used. As expected, isoproterenol increased the LVISP, but this positive inotropic response was larger in the soybean oil group compared to the control group (Figure 1D).

**Figure 2:**
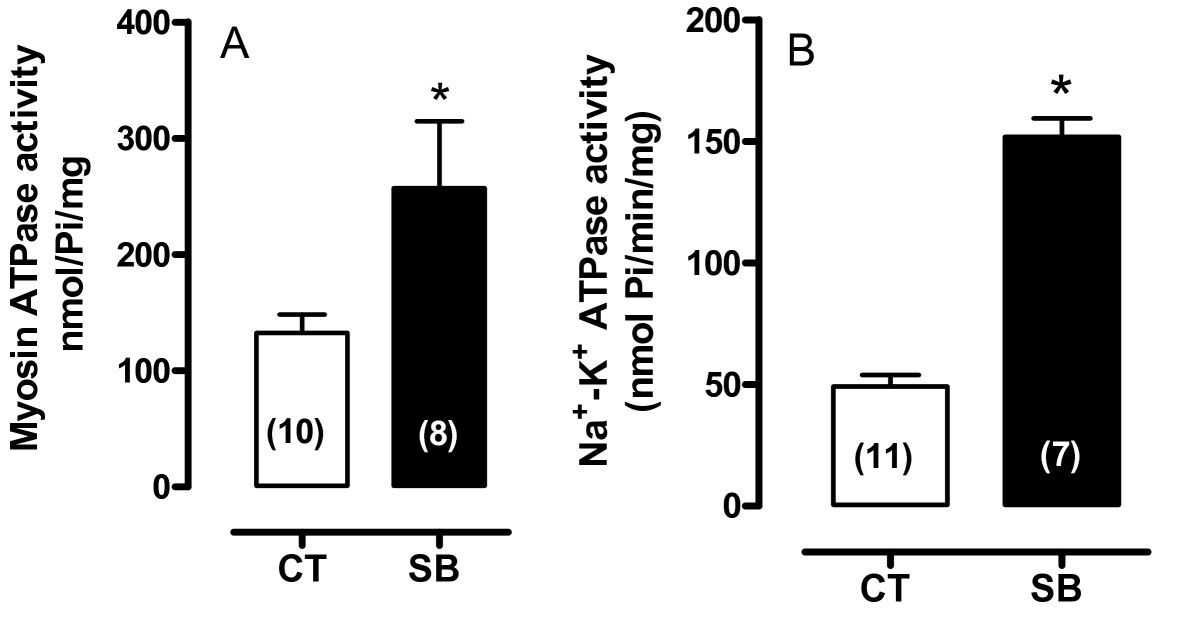
**Myosin ATPase activity (A) and Na**^**+**^**-K**^**+**^ **ATPase activity (B)**. Data represent mean ± SEM. *p < 0.05. Student "t" test. Number of animals used is indicated in parentheses.

**Figure 3:**
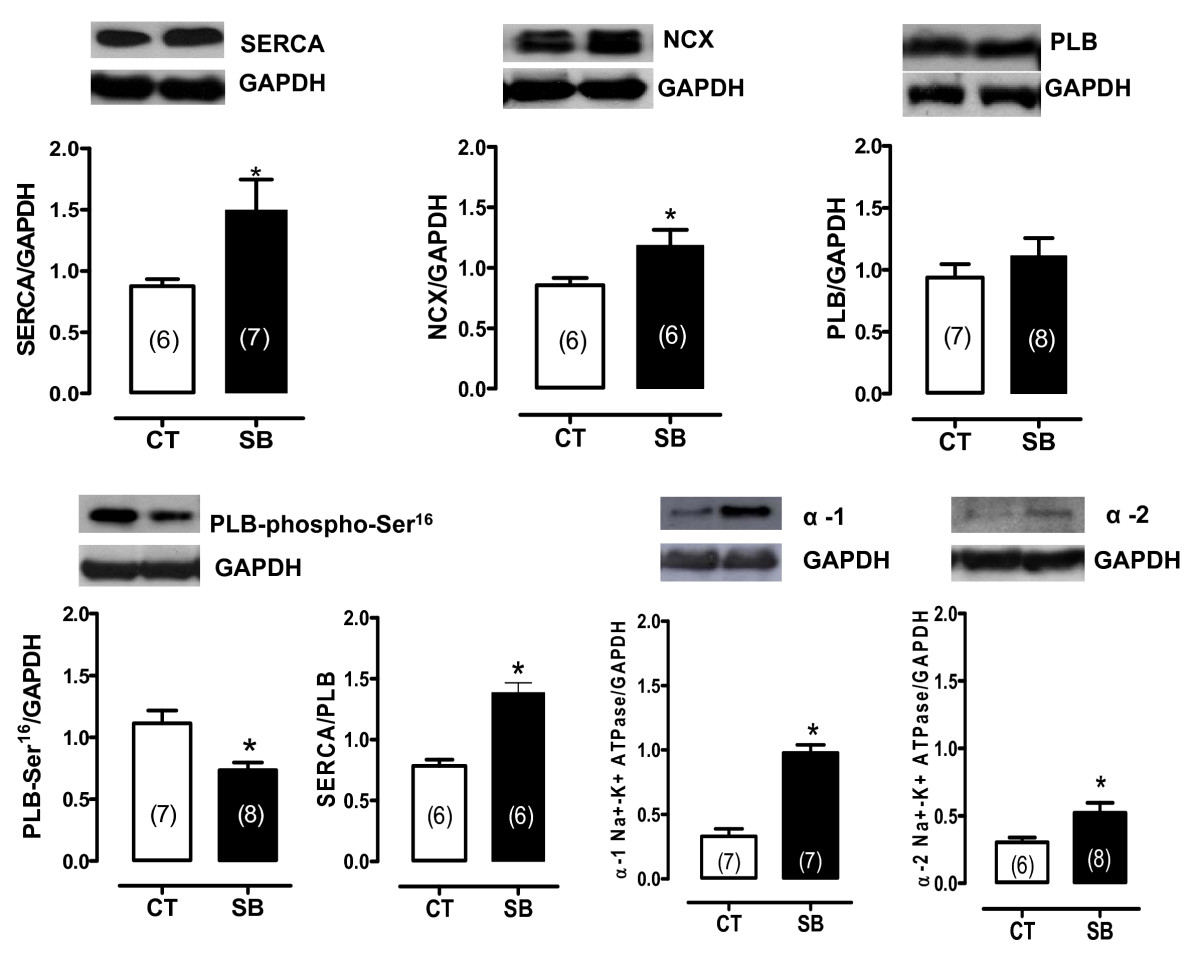
**Densitometric analysis of the Western blot for SERCA2a, sodium-calcium exchange (NCX), phospholamban (PLB), phosphorylated phospholamban (PLB-phospho-Ser**^**16**^**), SERCA/PLB ratio, isoforms α1 of Na**^**+**^**-K**^**+**^ **ATPase, and α2 of Na**^**+**^**-K**^**+**^ **ATPase protein expression in the hearts from control and SB-treated rats**. *p < 0.05 by Student's t-test. Number of animals used is indicated in parentheses. Representative blots are shown.

The potential effects of soybean oil treatment on the contractile machinery and other proteins involved in mechanical activity were also studied. Myosin and Na^+^-K^+^ ATPase activities were higher in the hearts of the soybean oil-treated group (Figure 2). Na^+^-K^+^ ATPase α1 and α2 isoforms, SERCA2a and NCX expression were also increased whereas PLB levels did not change (Figure 3). However, phosphorylated PLB was reduced even though the SERCA/PLB ratio was increased (Figure 3).

## Discussion

In this study, we demonstrated that treatment with soybean oil for 15 days is associated with an increased inotropic function of the left ventricle in an isolated perfused heart. This treatment did not cause cardiac hypertrophy but increased myosin, Na^+^-K^+^ ATPase activities, SERCA2a and NCX expression. Although PLB expression levels did not change, its phosphorylation was reduced. Also, in the present study, the soybean oil treatment did not affect blood pressure, but decreased the heart rate. Although SB-treated rats presented an increased inotropic response in isolated heart preparations, they did not exhibit changes in arterial or ventricular pressures. These results suggested that, in vivo, other mechanisms blunt the positive inotropism induced by soybean oil treatment. The reduction in heart rate suggests that reflex mechanisms might be involved.

The intrinsic, Frank-Starling mechanism, and extrinsic, β-adrenergic, inotropic responses evaluated *in vitro* showed an increment after treatment with soybean oil suggesting better myocardial performance. These findings are interesting as they were not associated with an increase in arterial blood pressure. A putative explanation for the better ventricular performance of the isolated hearts might be the increased myosin ATPase activity, NCX and SERCA2a expression, which are related to positive inotropism and regulation and maintenance of sarcoplasmic reticulum calcium load [10–12]. These findings might explain previous in vitro results [13, 14]. The sarcoplasmic reticulum is essential to regulate cytosolic calcium during myocardial contraction and relaxation. Sarcoplasmic reticulum calcium reuptake rate is regulated by the SERCA2a capacity. SERCA2a activity is regulated by a small molecular weight protein, PLB that can be phosphorylated at the serine 16 residue via the β-adrenergic pathway and the threonine 17 residue primarily via the calcium/calmodulin kinase II [15]. The phosphorylation status of PLB (p-PLB) has been related to the β-adrenergic signaling pathways [15]. Phosphorylation at either site is sufficient to remove the inhibitory effect of PLB on SERCA2a [16] and results in increased SR-Ca^2+^ uptake with enhanced myocyte contractility and relaxation. Our finding that PLB phosphorylated at serine^16^ was significantly reduced without any affect on PLB protein expression suggests a reduction in PKA-dependent PLB phosphorylation or an increase in dephosphorylated PLB [17] upon soybean oil treatment. A reduction in PKA-dependent PLB phosphorylation was shown to be dependent on changes in various levels of β-adrenergic signaling [18]. However, the present study demonstrated that even though p-PLB was reduced, SERCA2a protein expression was increased, thus resulting in a higher SERCA2a to PLB ratio. Indeed, previous report [19] suggests that the enrichment in docosahexaenoic acid (DHA) in cardiomyocytes phospholipids exerts positive influence on the β-adrenergic transduction mechanism, essentially through an increase of cAMP efficiency. The amount of DHA could be incorporated in cardiac membranes following fish or soybean oil diets. Therefore, we could speculate that the treatment with soybean oil could increase the amount of DHA in cardiomyocytes membranes phospholipids, which, in turn, could explain the increased β-adrenergic response in the soybean group.

The most well described disturbances in myocardial contractility are intimately dependent on the active transport of ions, namely Ca^2+^ and Na^+^, which are regulated by SERCA2a and the Na^+^-K^+^ ATPase. The Na^+^-K^+^ ATPase is a heteromeric protein consisting of α and βα subunits. While the α subunit contains the amino acids involved in catalytic function, ion transport and cardiac glycoside binding, the function of the β subunit, although not yet fully understood, is essential for normal activity of the enzyme and is involved in transport of the functional Na^+^-K^+^ ATPase in the plasma membrane [20]. The results of the present study demonstrate that soybean oil treatment increased expression of the α_1_ and α_2_ Na^+^-K^+^ ATPase protein subunits and its enzymatic activity. We speculate that this could be a cellular adaptation due to chronically higher intracellular Na^+^. This hypothesis is based on the results of a previous study in aortic smooth muscle cells [21]. These authors suggested that Na^+^-induced upregulation of the Na^+^-K^+^ ATPase α isoforms could be interpreted as an attempt by the cell to increase membrane Na^+^ transport to remove excess Na^+^ and restore it to normal intracellular levels. In cardiac myocytes, since an increase in Na^+^ concentration is associated with an increase in the intracellular Ca^2+^ concentration, the latter may contribute to the positive inotropic effect of soybean oil we observed. More recently a study have demonstrated that activation of the Na^+^-K^+^ ATPase modulates cardiac L-type Ca^2+^ channel function, leading to elevated sarcoplasmic reticulum Ca^2+^ release and intracellular Ca^2+^ transients [22]. These data led to a new mechanism that could explain the increased activity of the Na^+^-K^+^ ATPase associated with a positive inotropic effect of soybean oil treatment. However, to confirm our hypothesis the intracellular Na^+^ and Ca^2+^ need to be measured.

In conclusion, soybean oil treatment for 15 days in rats, although not affecting arterial pressure and LVSP, causes an increase in the left ventricular performance in the isolated heart. These changes were associated with increased SERCA2a expression and myosin ATPase activity. These findings are relevant because they help to clarify some of the cardioprotective mechanisms induced by soybean oil.

## Authors’ original submitted files for images

Below are the links to the authors’ original submitted files for images. Authors’ original file for figure 1 Authors’ original file for figure 2 Authors’ original file for figure 3
