# Supplementary figures and images for: Soybean oil increases SERCA2a expression and left ventricular contractility in rats without change in arterial blood pressure
Source: Lipids Health Dis. 2010 May 26;9:53. doi: 10.1186/1476-511X-9-53 (PMC2894821; doi:10.1186/1476-511X-9-53)

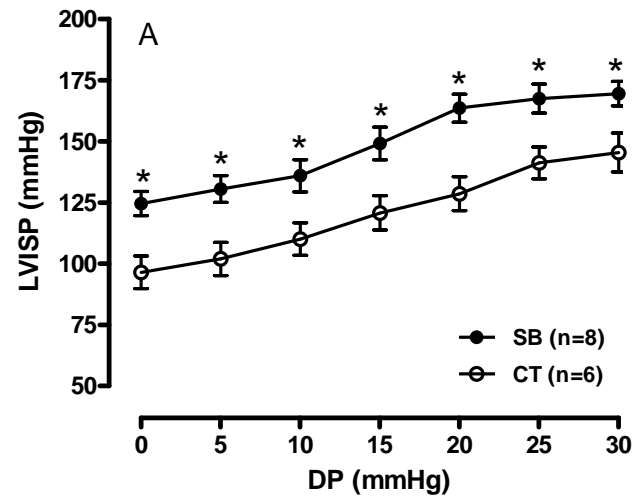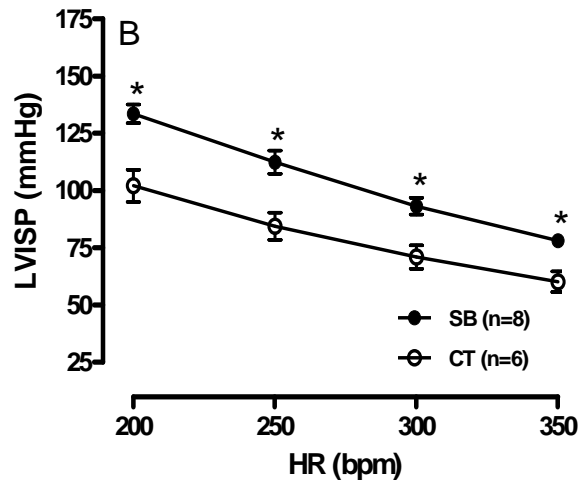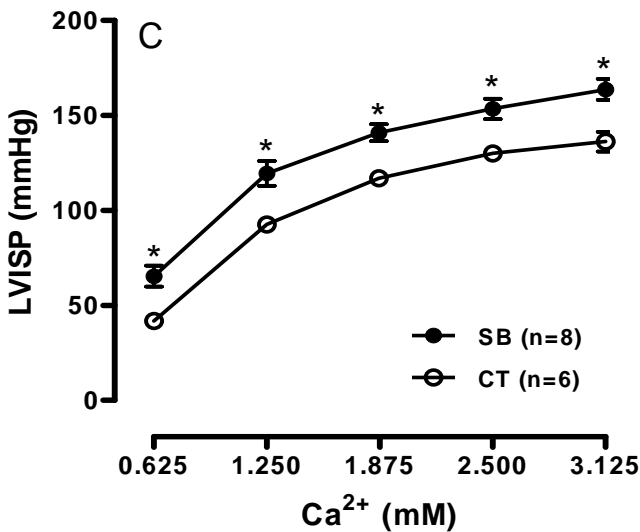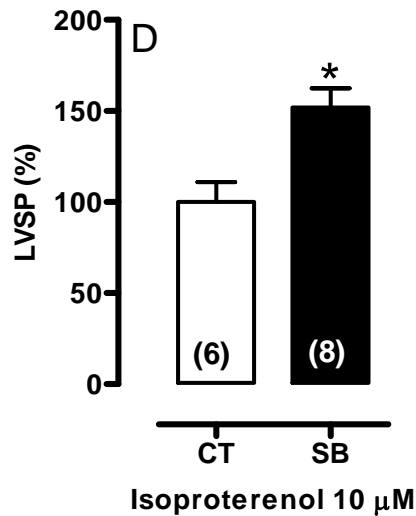

Supplement: Supplementary file 1 — Authors’ original file for figure 1 [file 12944_2010_302_MOESM1_ESM.pdf]

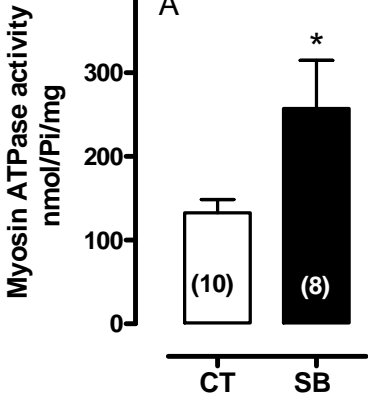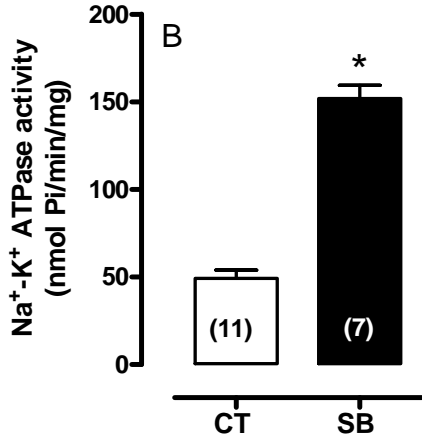

Supplement: Supplementary file 2 — Authors’ original file for figure 2 [file 12944_2010_302_MOESM2_ESM.pdf]

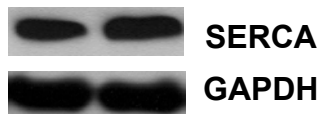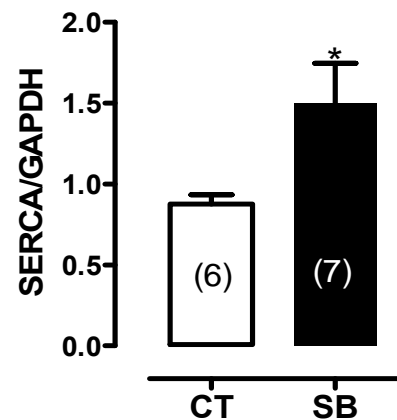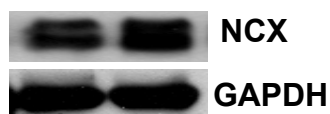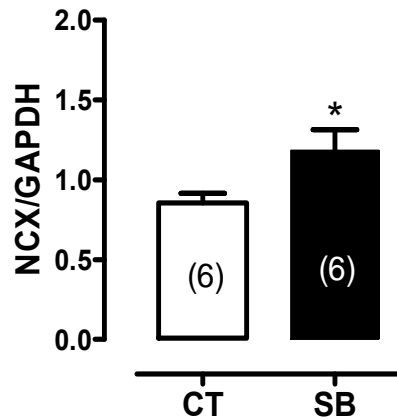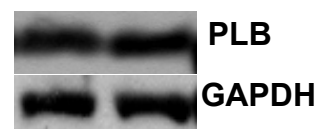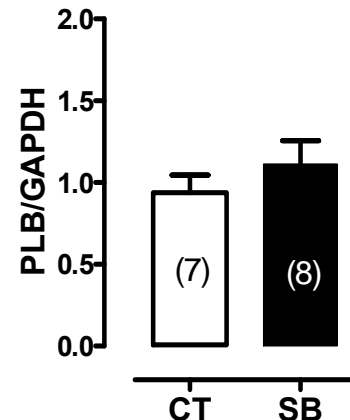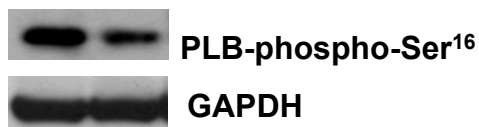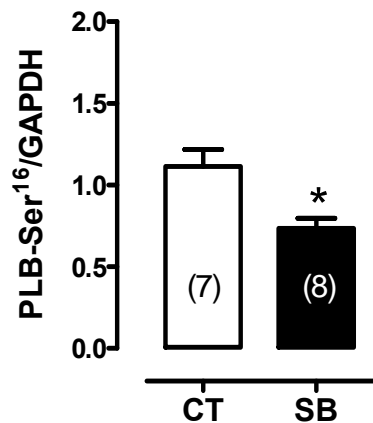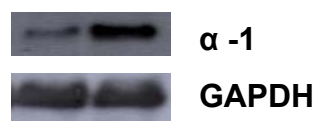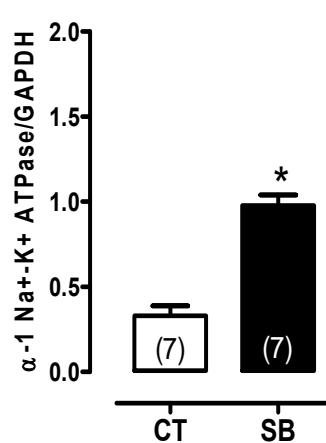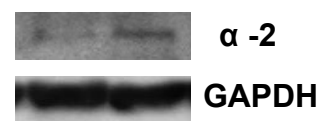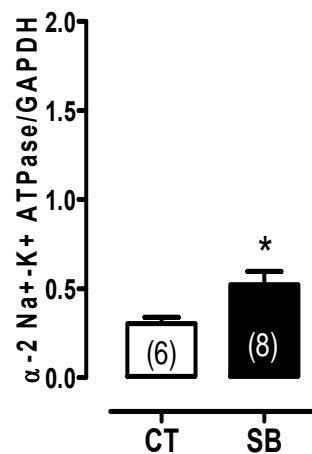

Supplement: Supplementary file 3 — Authors’ original file for figure 3 [file 12944_2010_302_MOESM3_ESM.pdf]
